# Genetic Associations of *PPARGC1A* with Type 2 Diabetes: Differences among Populations with African Origins

**DOI:** 10.1155/2015/921274

**Published:** 2015-04-21

**Authors:** Amanpreet K. Cheema, Tan Li, Juan P. Liuzzi, Gustavo G. Zarini, Mehmet T. Dorak, Fatma G. Huffman

**Affiliations:** ^1^Department of Dietetics and Nutrition, Robert Stempel College of Public Health, Florida International University, 11200 SW 8th Street, Miami, FL 33199, USA; ^2^Department of Biostatistics, Robert Stempel College of Public Health, Florida International University, 11200 SW 8th Street, Miami, FL 33199, USA; ^3^School of Health Sciences, Liverpool Hope University, Hope Park, Liverpool L16 9JD, UK

## Abstract

The aim of this study was to assess the differences in correlation of* PPARGC1A* polymorphisms with type 2 diabetes (T2D) risk in adults of African origins: African Americans and Haitian Americans. The case-control study consisted of >30 years old, self-identified Haitian Americans (*n* = 110 cases and *n* = 116 controls) and African Americans (*n* = 124 cases and *n* = 122 controls) living in South Florida with and without T2D. Adjusted logistic regression indicated that both SNP rs7656250 (OR = 0.22, *P* = 0.005) and rs4235308 (OR = 0.42, *P* = 0.026) showed protective association with T2D in Haitian Americans. In African Americans, however, rs4235308 showed significant risk association with T2D (OR = 2.53, *P* = 0.028). After stratification with sex, in Haitian Americans, both rs4235308 (OR = 0.38, *P* = 0.026) and rs7656250 (OR = 0.23, *P* = 0.006) showed protective association with T2D in females whereas in African American males rs7656250 had statistically significant protective effect on T2D (OR = 0.37, *P* = 0.043). The trends observed for genetic association of* PPARGC1A* SNPs, rs4235308, and rs7656250 for T2D between Haitian Americans and African Americans point out differences in Black race and warrant replicative study with larger sample size.

## 1. Introduction

Peroxisome proliferator activated receptor, gamma, coactivator 1 alpha (*PPARGC1A*) gene encodes a well-known protein, PGC-1*α* [1–5]. PGC-1*α* interacts with a wide array of nuclear receptor factors (NRFs) that further regulate several mitochondrial genes responsible for maintaining energy metabolism, mitochondrial function, and biogenesis [1–5]. In addition, PGC-1*α* regulates fatty acid oxidation as well as oxidative phosphorylation by interaction with peroxisome proliferative activated receptor alpha (*PPARA*) and estrogen receptor-related receptor (ESRR) [1–6]. Upregulation of glucose transporter-4 (GLUT-4) by PGC-1*α* increases glucose uptake in skeletal muscle cells and increases phosphoenolpyruvate carboxy-kinase and glucose-6-phosphatase activities [7, 8]. This versatility of PGC-1*α* as a master coactivator of various metabolic processes has put it on a center stage for variety of human metabolic diseases such as type 2 diabetes (T2D) [9].

Reduced expression of PGC-1*α* has been reported not only in individuals with T2D, but also in individuals who are unaffected, who have a family history of T2D [10]. Ethnic heterogeneity observed in genetic associations of* PPARGC1A* polymorphisms with T2D could be due to the presence of causal or other polymorphisms in strong linkage disequilibrium (LD) with the polymorphism in question [11–14]. Differences in LD or gene to gene interactions among ethnicities could also be a possible explanation for such observed differences. Moreover, the environment in which populations live varies around the world. This variation in the interaction of environment with gene of interest could also be instrumental in different associations of* PPARGC1A* polymorphisms with T2D across ethnicities.

Differences in genetic variations and environmental factors (diet, lifestyle, and physical inactivity) between ethnicities have in fact been identified to be associated with T2D [11–14]. Compared to non-Hispanic Whites, the risk of T2D is 77% higher among non-Hispanic Blacks [15]. Although the adipogenic diet puts African Americans at high risk for T2D, the role of genetics cannot be ruled out. African Americans received “thrifty gene” from their African ancestors that helped them survive in case of unavailability of food [16]. The “thrifty gene” along with diet with poor nutrition has made African Americans the high risk population for T2D [16]. Quite often, the lines that separate various subpopulations within the “Black” community are blurred in research studies, which make association studies difficult, due to presence of genetic heterogeneity within the sample. Haitian Americans are generally grouped together with other populations of African origins but the prevalence of T2D in Haitian Americans is nowhere close to African Americans [17]. Apart from African descent, populations in Haiti also have lineage from France and Spain making them unique [18]. In 2010, the International Diabetes Federation estimated the T2D prevalence in Haiti to be 7.2% for 20 to 79 year olds [17] yet the official data for Haitian Americans are not available. The latest US Census Bureau data (2008) indicates the presence of 546,000 Haitian immigrants in the United States, 46% of total Haiti-born population resides in Florida, and more specifically 34.2% reside in the Miami-Dade and Broward Counties, FL [19]. Therefore, genetic association studies are important for* PPARGC1A* gene, which is implicated in energy metabolism and T2D in populations with African origins. However, there is lack of data on the relationship between* PPARGC1A* polymorphisms and T2D outcomes in Haitian Americans. Therefore, the principle focus of this study was to investigate the differences in genetic association of* PPARGC1A* polymorphism with phenotype such as T2D between Haitian American and African American adults residing in south Florida.

## 2. Materials and Methods

### 2.1. Study Population

Self-identified Haitian Americans and African Americans living in South Florida, ages >30 years, were recruited at the Human Nutrition Laboratory, Department of Dietetics and Nutrition, Robert Stempel College of Public Health and Social Work, Florida International University, for a case control cross-sectional study. Recruitment of participants was done using invitational flyers, community-based sources, and advertisements in English and Creole. The presence of T2D was self-reported by the participants and was confirmed with laboratory tests using American Diabetes Association criteria (fasting plasma glucose concentration ≥126 mg/dL or use of insulin or diabetes medication). Individuals with any other chronic condition, pregnancy or lactation, were excluded from the study. The research purpose and protocol were explained in English as well as Creole to the participants and voluntary informed consent was procured. Institutional Review Board (IRB) approval was received from Florida International University prior to study initiation.

### 2.2. Sociodemographics, Anthropometrics, and Medical Assessment

The information on demographics such as age, gender, T2D medication use, and smoking history was collected using questionnaire to match cases and controls for both ethnicities by trained research staff. Height as well as weight were measured using SECA balance scale (Seca Corp., USA). Body mass index (BMI) was then calculated in kg/height in m^2^. A nonstretchable measuring tape measured waist circumference (WC) to the nearest 0.1 cm by placing it midway between the 12th rib and iliac crest at minimal respiration. After 15-minute rest, sphygmomanometer (Tycos 5090-02 Welch Allyn Pocket Aneroid Sphygmomanometer, Arden, NC, USA) and a stethoscope (Littmann Cardiology, 3M, St. Paul, MN, USA) were used to measure blood pressure (BP).

### 2.3. Blood Collection and DNA Isolation

Twenty mL of venous blood was collected from each individual after an overnight fast (at least 8 hours) by a certified phlebotomist using standard laboratory techniques. Genomic DNA was then isolated from the whole blood using QIAamp DNA Blood Mini Kit (Qiagen, Hilden, Germany), according to the vender's recommended protocol. Quality and quantity of the isolated DNA were tested using 2000c nanodrop spectrophotometer (Thermo Scientific, USA).

### 2.4. Single Nucleotide Selection and Genotyping

**Figure 1:**
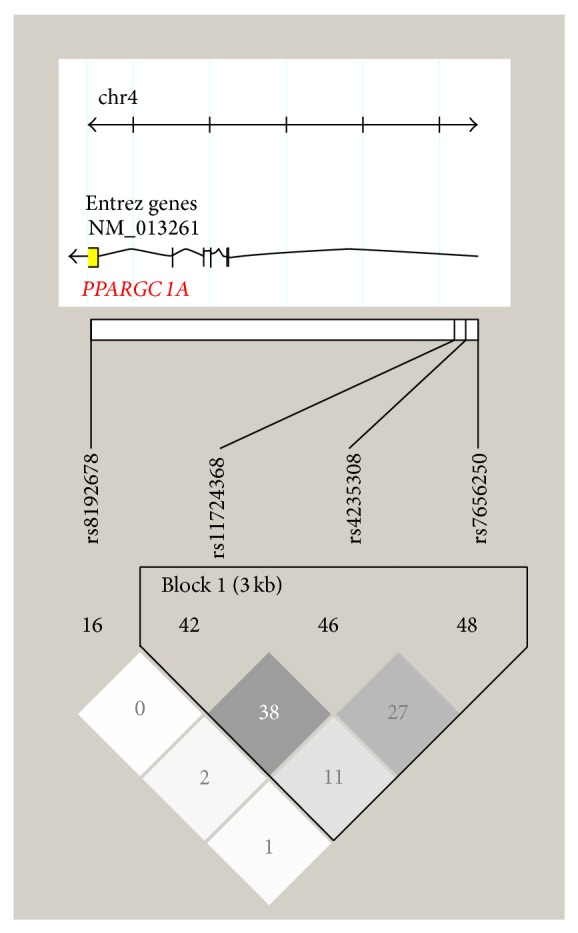
Haploview plot showing linkage disequilibrium (LD) with *r* ^2^ values for four selected SNPs of* PPARGC1A* gene. Note: black coloring displays strong LD, dark grey displays less strong LD, light grey displays intermediate LD, and white displays weak LD.

The* PPARGC1A* gene is located in 4p15.1 region spanning ~110 kb. The rationale behind SNP selection was to give equal emphasis to functionality, already known disease associations, statistical power, and cost. The four SNPs were selected for genotyping (Table 1) using HapMap (http://www.hapmap.org/) genotype data from Africans, taking into account their relationships with each other. These SNPs were tested for interrelationships using linkage disequilibrium (LD) plots. TAGGER on Haploview was used for selection of haplotype tagging SNPs. The independence of each SNP from others is evident in the LD plot (Figure 1). The values shown in this plot are *r* ^2^ values showing the correlation between any pair of SNPs. The highest *r* ^2^ value for any pairwise comparison for the four selected SNPs is 0.38 as shown in Figure 1. An integrated selection on the basis of genetic associations and human genome epidemiology was done using HuGE Navigator and dbSNP. Functionality of SNPs was assessed bioinformatically on F-SNP website (http://compbio.cs.queensu.ca/F-SNP/). Thus, seventy-five SNPs were narrowed down using mathematical, biological, and bioinformatics approach to four that have high minor allele frequencies (MAF), robust disease associations, high functionality, and no correlation with one another.

The main characteristics for the selected* PPARGC1A* gene Single Nucleotide Polymorphisms (SNPs) genotyped are shown in Table 1. Genotyping for all four SNPs was performed by real-time PCR amplification on BioRad CFX96 real-time PCR instrument (Hercules, CA) using commercially available TaqMan allelic discrimination assays (LifeTech, Foster City, CA). PCR amplification (20 *μ*L) was performed in 96-well plates using Bio-Rad SsoFast Probes Supermix as the reaction buffer with the TaqMan Assay. To ensure reproducibility and reliability of genotyping method, 10% of the DNA samples were duplicated during genotyping. Bio-Rad CFX Manager software (version 3.0) was utilized for both data acquisition and assignment of genotypes for each SNP.

### 2.5. Statistical Analysis

The statistical analyses were done using SPSS version 20 (SPSS Inc., Chicago, IL, USA). All statistical tests were two-tailed, and the threshold for statistical significance was set at *P* ≤ 0.05. Sample size calculation was performed prior to the initiation of the study. Sample size of *n* = 62 was calculated for significance threshold of 0.05 and odds ratio of 1.5 for equal case and control, to have statistical power of 80%. Genotype counts in each SNP were checked for Hardy-Weinberg equilibrium (HWE) in controls using the Chi-squared goodness-fit test. Demographic and clinical information between cases and controls was compared using Student's *t*-test for continuous variables and Chi-squared test for categorical variables. All genetic associations were assessed by using the recessive genetic model to detect recessive effects, often overlooked by other genetic models. Logistic regression methods were used to calculate unadjusted and adjusted odds ratios (OR) and 95% confidence intervals (CIs) to assess the relationship of all SNPs simultaneously with binary outcome for case-control status (T2D = Yes/No) before and after adjusting for potential confounding factors such as age, sex, smoking status, and BMI. The analysis also included interaction term for SNPs and sex. Due to heterogeneity among two ethnicities, these two groups were analyzed separately. Stratified analysis by ethnicity and sex was performed, to assess their effect modification on the relationship of polymorphisms with the phenotype, that is, T2D. The analysis was then repeated adjusting for age, BMI, and smoking status. The multiple linear regression analysis was employed to test the association of insulin plasma concentration and the presence of polymorphisms in controls of both Haitian American and African American participants separately. The analysis was adjusted with confounders: age, sex, BMI, and smoking status. The insulin values were log transformed before analysis.

## 3. Results

A total of 226 Haitian Americans (*n* = 110 cases, *n* = 116 controls) and 246 African Americans (*n* = 124 cases and *n* = 122 controls) comprised the study population for his study.

### 3.1. General Characteristics

Table 2 shows the general characteristics of the individuals in the study. In brief, individuals with T2D (cases) were older than those without T2D (controls) in both Haitian Americans (*P* = 0.001) and African Americans (*P* = 0.022). Cases in Haitian American (*P* = 0.019) as well as African American group (*P* < 0.001) had higher waist circumference than controls. However, BMI was significantly higher for cases as compared to controls in African Americans only (*P* < 0.001). There was no significant difference between cases and controls in Haitian American group for either SBP or DBP, whereas, SBP was significantly higher in cases as compared to controls in African American group (*P* = 0.006).

The cases in Haitian American group included 48 males (44%) and 62 females (56%) and the controls included 54 males (47%) and 62 females (53%). The cases in African American group constituted 59 males (48%) and 65 were females (52%). The African American controls comprised of equal males and females (*n* = 61, 50%).

### 3.2. Frequency of* PPARGC1A* Polymorphisms

All cases and controls were genotyped for the four candidate SNPs. Genotype call rates were higher than 95% for cases and controls in both ethnicities. None of the four* PPARGC1A* SNPs showed any deviation from Hardy-Weinberg equilibrium in controls.  Table 3 shows genotype distribution of all four* PPARGC1A* SNPs in the case-control sample for both ethnicities. The minor allele frequency (MAF) for rs8192678, rs7656250, rs4235308, and rs11724368 SNP was 0.145 and 0.060; 0.118 and 0.090; 0.414 and 0.327; 0.072 and 0.069 for cases and controls of Haitian Americans, respectively. In African American group, the MAF for rs8192678, rs7656250, rs4235308, and rs11724368 for cases and controls was 0.093 and 0.074; 0.165 and 0.110; 0.343 and 0.336; 0.093 and 0.069, respectively (Table 3). The MAF seen in the study was very close to NCBI's genotyped data validating our study (http://www.ncbi.nlm.nih.gov/SNP/).

### 3.3. Correlations between* PPARGC1A* Polymorphisms and Type 2 Diabetes

In total, four* PPARGC1A* SNPs were examined simultaneously for their genetic associations with T2D using logistic regression analysis. Results including unadjusted odds ratios and odds ratios adjusted for covariates (age, sex, BMI, and smoking status) and interaction terms between SNPs and sex are shown in Tables 4(a) and 4(b). Two out of four SNPs showed significant association with T2D in Haitian Americans. However, only one SNP was significantly associated with T2D in African Americans (Table 4(b)). The SNP rs7656250 showed protective association with T2D with adjusted OR of 0.22 (*P* = 0.005) in Haitian Americans (Table 4(a)). This association was not significant for African American group but when adjusted for confounders, rs7656250 showed risk association with T2D with OR of 1.02 (*P* = 0.940) though it did not reach statistical significance (Table 4(b)). The interaction between sex and rs7656250 was found to be significant only in Haitian Americans (*P* = 0.008). In Haitian Americans, rs4235308 had an unadjusted odds ratio (OR) of 0.53 (*P* = 0.033) as shown in Table 4(a). The adjustment for age, BMI, sex, smoking, and interaction terms for SNPs and sex lowered the effect (OR = 0.42, *P* = 0.026). This SNP showed significant risk association with T2D in African Americans (OR = 2.53, *P* = 0.028) (Table 4(b)).

Effect modification of sex on* PPARGC1A* SNPs association on T2D was also explored by stratification by sex adjusted for age, BMI, and smoking status, as shown in Tables 5(a) and 5(b). In Haitian Americans, rs4235308 showed protective association with T2D both in females (OR = 0.38, *P* = 0.026) and in males (OR = 0.62, *P* = 0.326), though not statistically significant. In Haitian Americans, rs7656250 also had a protective effect on T2D in females (OR = 0.23, *P* = 0.006) and but risk association in males (OR = 1.62, *P* = 0.409). The association in males was statistically insignificant. In African American females, rs7656250 showed risk association though statistically nonsignificant (OR = 1.14, *P* = 0.788), whereas in males, it had statistically significant protective effect on T2D (OR = 0.37, *P* = 0.043). In African American females, rs4235308 had stronger risk association with T2D (OR = 2.69, *P* = 0.029) but not in males (OR = 1.16, *P* = 0.723).

The association of insulin plasma concentration and presence of these polymorphisms in either Haitian American or African American controls was analyzed. The results in Haitian Americans were not significant for any of the SNPs (results not shown). However, in African American controls, the presence of rs4235308 C allele (CC + CT) increased the likelihood of higher log insulin by 0.140 times than those with TT genotype (*P* = 0.008). All other SNPs showed no significant association with log insulin.

## 4. Discussion

High prevalence of T2D in populations with African origins is well established [20–22]. Recently, only few studies have documented existing metabolic differences in the subpopulations of African ancestry [23–25]. Despite being well established, the ethnic disparity is not always addressed in genetic association studies. There exists an assumption that ethnic groups within a race are homogenous and obvious differences among different members of the ethnic group and many times subgroups with the ethnicity are overlooked. This study revealed such differences among Haitian Americans and African Americans, often grouped together with other populations of African origins. This study also provides some confirmation of minor allele frequencies of previously discovered genetic markers associated with T2D, furthermore validating our case-control study.

Of the four* PPARGC1A* SNPs, rs4235308 showed significant overall association with T2D, while rs8192678, rs7656250, and rs11724368 did not show any associations in African American group. However, in Haitian American group, both rs7656250 and rs4235308 showed overall association. Ling et al. (2008) reported association of reduced* PPARGC1A* mRNA expression with rs8192678 SNP, making some to speculate it as a functional SNP [26]. The association of rs8192678 SNP with T2D has also been reported in Danish [27], Japanese [28], Southern Chinese [29, 30], and North Indians [31], but no such association was reported in Pima Indians [32] or in French Caucasians [33]. These discrepancies in genetic associations in different populations could merely be due to different genetic admixture or due to errors in sampling, low statistical power, population not being homogenous, confounding by gene-environment interactions, and stringency for genome wide studies (GWAS). On the other hand, these conflicting results suggest ethnic differences in distribution of the SNPs in different populations and thus differences in susceptibility for T2D in various ethnicities. It is often seen that a genetic association is rather with a nearby SNP than the SNP being tested due to confounding by locus. We made sure that the SNPs selected for the study were independent and the associations were not due to linkage disequilibrium between these gene variants.

An interesting finding in the study was the protective association of rs7656250 as well as rs4235308 with T2D in Haitian Americans whereas risk association observed for rs4235308 in African Americans. Further, we found the association of rs4235308 SNP with higher log insulin values in African American controls. The findings could partly explain the prevalence of insulin resistance in African Americans, which is a powerful predictor of T2D. The genetic implication of these polymorphisms on insulin resistance in African Americans could also bolster results from a previous study reporting the ethnic differences in insulin resistance and other indicators of glucose metabolism among Haitian Americans and African Americans [25]. Further, a risk association was observed for SNPs rs7656250 and rs4235308, in females of African Americans in the study, whereas, both rs7656250 as well as rs4235308 exhibited protective effect in females of Haitian American group. Haitian Americans have poor diabetes control but lower prevalence than African Americans [34]. The collective protective effect of* PPARGC1A* polymorphisms rs7656250 and rs4235308 in this study in Haitian Americans could be just a glimpse of why such a difference exists. One study pointed out the differences between both ethnicities of South Florida in diet quality [35]. Although both ethnicities were found to have lower than optimal diet quality, Haitian Americans had better diet quality scores in general but not in women [35]. The prevalence of T2D has been reported to be higher in Haitian females than males in one study [36] although the study population is comprised of only the members of the households present at the time of the visit. This selection bias could have resulted in overestimation of diabetes prevalence in females. Additionally, the gender differences in prevalence of T2D in Haitian Americans are not well known due to lack of literature. The poor access to health care, educational status, exposure to gestational diabetes, and diet quality often seen in ethnicities of African origins may increase the lifestyle burden on physiological functioning and thereby increasing prevalence of T2D in females [37]. According to a recent study published in Journal of American Medical Association, African American females had 2.4-fold greater diabetes incidence per 1000 person as compared to 1.5-fold greater in men than their White counterparts [38]. The strong risk association for rs4235308 in African American females observed in this study follows the trend. However, the risk association of rs7656250 in African Americans could not reach statistical significance, probably due to insufficient sample size. In African males, the association of rs7656250 was marginally protective for T2D; probably it can explain why African American males have lower T2D prevalence than African American females. As there is lack of genetic association studies in African American population and virtually nonexistent in Haitian American population, further research is warranted.

There are few limitations of this study. Although, the sample size of the study had sufficient statistical power (>80%) to detect odds ratio of 1.5 or more, for equal case and control at significance threshold of 0.05, it may have been inadequate to detect association of SNPs with a modest effect. As with any case-control approach, bias exists for genetic association studies, due to unacceptable designation of cases and controls. In this study, participants were classified as cases or controls (T2D = Yes/No) with the use of medical history and the standard criteria described by American Diabetes Association. Self-reported ethnicity is a common method with population based association studies and due to population stratification it may increase the false positive results. In this study, both cases and controls were selected from the same population pool and geographic area, with information on ethnicity up to two generations, for each respective ethnicity. The heterogeneity however within the African American and Haitian American population and thus residual confounding is still a concern.

Despite the low *P* values, the likelihood of true disease associations mostly depends on the biological plausibility. Polymorphisms located within the* PPARGC1A* gene with strong associations with T2D have been reported in multiple genetic association studies [39–43]. The* PPARGC1A* gene has been identified as a transcriptional coactivator of a series of nuclear receptors, which regulate processes that impact cellular energy metabolism, thermogenesis regulation, glucose metabolism, adipogenesis, and oxidative metabolism via protein PGC-1*α* [4, 44, 45]. Acetylation of PGC-1*α* is in fact essential for its transcriptional coactivator functions [46] and any hindrance in acetylation-deacetylation process may adversely affect its functioning. PGC-1*α* dysregulation is often associated with insulin resistance and T2D [47], which suggest that variations within the* PPARGC1A* gene may influence transcriptional homeostasis of the genes involved.

## 5. Conclusions

In summary, this is the only study that successfully examined differences in genetic associations of* PPARGC1A* with T2D between Haitian American and African Americans. As T2D is a complex disease with strong environmental influence, the contribution of differences in ancestry may be behind the ethnic disparities observed in risk of type 2 diabetes development in this and other populations.

## Figures and Tables

**Table 1:** Characteristics of *PPARGC1A* SNPs.

NCBI ref SNP number^*^	Chromosome nucleotide position^‡^	MAF^†^	Disease risk associations	*F*-score
rs8192678	23815662	0.291	T2D, CVD, Obesity	0.50
rs7656250	23866016	0.265	T2D, CVD	0.27
rs4235308	23864412	0.396	CVD	0.28
rs11724368	99418507	0.106	CVD	0.25

Note: ^*^National Center for Biotechnology Information (NCBI) reference single nucleotide polymorphism (SNP) number (http://www.ncbi.nlm.nih.gov/).

^‡^Genome Reference Consortium Human Build 37 patch release 13 (GRCh37.p13) used for nucleotide position (http://www.ncbi.nlm.nih.gov/SNP/).

^†^Minor allele frequencies are from a global population genotyped in HapMap project.

MAF: minor allele frequency; T2D: type 2 diabetes; CVD: cardiovascular disease; *F*-Score: functionality score.

**Table 2:** Descriptive characteristics of individuals by ethnicity and T2D status.

Variables	Haitian Americans			African Americans		
	Cases (*n* = 110)	Controls (*n* = 116)	*P* value	Cases (*n* = 124)	Controls (*n* = 122)	*P* value
Age, yr.	58.55 ± 10.15	54.03 ± 11.05	0.001	54.31 ± 10.07	51.20 ± 8.65	0.022
Sex (male)	48 (44)	54 (46)	0.484	59 (48)	61 (50)	0.704
Waist circumference (cm)	100.25 ± 12.16	95.97 ± 12.72	0.019	114.15 ± 18.12	102.02 ± 14.98	<0.001
BMI (kg/m^2^)	29.50 ± 5.45	28.96 ± 5.157	0.628	35.86 ± 8.28	31.21 ± 6.77	<0.001
Smoke (Yes)	7 (6)	5 (4)	0.490	44 (35)	49 (40)	0.532
Blood pressure (mm of Hg)						
SBP	148.24 ± 25.76	144.63 ± 26.206	0.276	140.85 ± 20.11	133.15 ± 18.41	0.006
DBP	90.82 ± 13.22	90.44 ± 13.55	0.853	89.76 ± 11.59	88.37 ± 12.97	0.399
Diabetes meds (Yes)	98 (89)	0 (0)	NA	96 (77)	0 (0)	NA

Note: values are unadjusted mean ± SD for continuous variables or *N* (%) for categorical variables. Diabetes medication is only for cases. So statistical test is not necessary and the *P* value is not available (NA). Cases: with T2D; controls: without T2D; BMI: body mass index; diabetes meds: diabetes medications; SBP: systolic blood pressure; DBP: diastolic blood pressure.

**Table 3:** Genotype distribution of *PPARGC1A* SNPs by ethnicity and T2D.

SNPs	Minor allele	Haitian Americans (*n* = 226)					African Americans (*n* = 246)				
		Cases (*n* = 110)	Controls (*n* = 116)	*P*-value	MAF (%)		Cases (*n* = 124)	Controls (*n* = 122)	*P* value	MAF (%)	
					Cases	Controls				Cases	Controls
Genotype frequencies (*n*, %)											
rs8192678											
CC		92 (84)	102 (88)				103 (84)	104 (85)			
CT	T	4 (4)	14 (12)	0.000	0.145	0.060	19 (15)	18 (15)	0.365	0.093	0.074
TT		14 (13)	0 (0)				2 (2)	0 (0)			
rs7656250											
TT		85 (73)	96 (83)				90 (72)	98 (80)			
CT	C	24 (22)	19 (16)	0.579	0.118	0.090	27 (22)	21 (17)	0.263	0.165	0.110
CC		1 (0.9)	1 (0.8)				7 (6)	3 (2)			
rs4235308											
TT		35 (32)	52 (45)				56 (45)	51 (42)			
CT	C	59 (54)	52 (45)	0.124	0.441	0.327	51 (41)	60 (49)	0.327	0.343	0.336
CC		16 (14)	12 (10)				17 (14)	11 (9)			
rs11724368											
CC		93 (84)	100 (86)				103 (83)	105 (86)			
CG	G	17 (15)	16 (14)	0.724	0.072	0.069	19 (15)	17 (14)	0.347	0.093	0.069
GG		0 (0)	0 (0)				2 (2)	0 (0)			

Note: genotype frequencies are depicted as *n* (%). Cases: with T2D; controls: without T2D; SNP: single nucleotide polymorphism; MAF: minor allele frequency.

**(a):** 

Variables		Haitian American							
		Unadjusted OR	95% CI		*P* value	Adjusted OR	95% CI		*P* value
rs8192678	TT + CT versus CC	0.66	0.30	1.42	0.285	0.49	0.15	1.60	0.228
rs7656250	CC + CT versus TT	0.66	0.34	1.30	0.231	**0.22**	**0.07**	**0.64**	**0.005**
rs4235308	CC + CT versus TT	**0.53**	**0.30**	**0.95**	**0.033**	**0.42**	**0.17**	**0.93**	**0.026**
rs11724367	CC + CG versus GG	1.14	0.52	2.52	0.745	1.73	0.55	5.49	0.353
rs8192678 ∗ sex	—	**—**	**—**	**—**	**—**	1.77	0.34	9.27	0.490
rs7656250 ∗ sex	—	**—**	**—**	**—**	**—**	**7.53**	**1.66**	**34.15**	**0.008**
rs4235308 ∗ sex	—	**—**	**—**	**—**	**—**	1.56	0.46	5.34	0.444
rs11724367 ∗ sex	—	**—**	**—**	**—**		0.54	0.09	2.95	0.483

Note: the statistically significant results are in bold. Controlled variables included in the logistic regression analysis for adjusted OR were age, sex, BMI, and smoking status. The interactions between sex and individual SNP were also included in logistic regression analysis for all the SNP. *P* is considered significant at 0.05. OR**:** odds ratio; CI: confidence interval; *PPARGC1A*: peroxisome proliferator activated receptor, gamma, coactivator 1 alpha.

**(b):** 

Variables		African American							
		Unadjusted OR	95% C.I.		*P*value	Adjusted OR	95% C.I.		*P* value
rs8192678	TT + CT versus CC	0.90	0.45	1.87	0.777	0.55	0.19	1.56	0.269
rs7656250	CC + CT versus TT	0.62	0.34	1.13	0.117	1.02	0.43	2.43	0.940
rs4235308	CC + CT versus TT	1.29	0.75	2.21	0.356	**2.53**	**1.08**	**5.92**	**0.028**
rs11724367	CC + CG versus GG	0.69	0.33	1.25	0.329	0.29	0.08	1.14	0.073
rs8192678 ∗ sex	—	—	—	—	—	1.46	0.38	5.60	0.585
rs7656250 ∗ sex	—	—	—	—	—	0.36	0.11	1.13	0.079
rs4235308 ∗ sex	—	—	—	—	—	0.48	0.15	1.59	0.220
rs11724367 ∗ sex	—	—	—	—	—	3.78	0.82	17.31	0.082

Note: the statistically significant results are in bold. Controlled variables included in the logistic regression analysis for adjusted OR were age, sex, BMI, and smoking status. The interactions between sex and individual SNP were also included in logistic regression analysis for all the SNP. *P* is considered significant at 0.05. OR**:** odds ratio; CI: confidence interval; *PPARGC1A*: peroxisome proliferator activated receptor, gamma, coactivator 1 alpha.

**(a):** 

Male									
Variables		Haitian American				African Americans			
		OR	95% CI		*P* value	OR	95% CI		*P* value
rs8192678	TT + CT versus CC	0.89	0.25	3.10	0.854	0.86	0.29	2.53	0.786
rs7656250	CC + CT versus TT	1.62	0.51	5.09	0.409	**0.37**	**0.14**	**0.97**	**0.043**
rs4235308	CC + CT versus TT	0.62	0.24	1.61	0.326	1.16	0.50	2.68	0.723
rs11724368	CC + CG versus GG	0.84	0.23	3.08	0.790	1.11	0.42	2.94	0.829

Note: the statistically significant results are in bold. Controlled variables included in the logistic regression analysis for OR (adjusted) were age, sex, BMI, and smoking status. OR: odds ratio; CI: confidence interval; *PPARGC1A*: peroxisome proliferator activated receptor, gamma, coactivator 1 alpha.

**(b):** 

Female									
Variables		Haitian American				African Americans			
		OR	95% CI		*P* value	OR	95% CI		*P* value
rs8192678	TT + CT versus CC	0.51	0.15	0.16	0.257	0.48	0.15	1.49	0.205
rs7656250	CC + CT versus TT	**0.23**	**0.08**	**0.65**	**0.006**	1.14	0.43	3.07	0.788
rs4235308	CC + CT versus TT	**0.38**	**1.59**	**0.89**	**0.026**	**2.69**	**1.11**	**6.52**	**0.029**
rs11724368	CC + CG versus GG	1.41	0.45	4.40	0.555	0.32	0.07	1.54	0.155

Note: the statistically significant results are in bold. Controlled variables included in the logistic regression analysis for OR (adjusted) were age, sex, BMI, and smoking status. OR: odds ratio; CI: confidence interval; *PPARGC1A*: peroxisome proliferator activated receptor, gamma, coactivator 1 alpha.

## Abbreviations

SNPs: Single nucleotide polymorphisms
*PPARGC1A*: Peroxisome proliferator activated receptor, gamma, coactivator 1 alpha
T2D: Type 2 diabetes
SD: Standard deviation
OR: Odds ratio
CI: Confidence interval.
